# Supplementary material for: Model-based stationarity filtering of long-term memory data applied to resting-state blood-oxygen-level-dependent signal
Source: PLoS One. 2022 Jul 27;17(7):e0268752. doi: 10.1371/journal.pone.0268752 (PMC9328502; doi:10.1371/journal.pone.0268752)
Supplement: S1 File — In this supplementary information file, we provide complementary information that aims to sharpen the intuition behind the usage of different matrices for analysing the effect of proposed filter on resting state BOLD signals. (PDF) [file pone.0268752.s001.pdf]

SI Text of "Model-based stationarity filtering of long-term memory data applied to resting-state blood-oxygen-level-dependent signal"

Ishita Rai Bansal[1], Arian Ashourvan[2,3], Maxwell Bertolero[2], Danielle S. Bassett[2,3,4,5,6,7], Sérgio Pequito [1*]

1 Delft Centre for Systems and Control, Delft University of Technology, Delft, Netherlands
2 Department of Bioengineering, School of Engineering and Applied Science, University of Pennsylvania, United States
3 Penn Center for Neuroengineering and Therapeutics, University of Pennsylvania, United States
4 Department of Neurology, Hospital of the University of Pennsylvania, United States
5 Department of Psychiatry, Perelman School of Medicine, University of Pennsylvania, United States
6 Department of Electrical & Systems Engineering, School of Engineering and Applied Science, University of Pennsylvania, United States
7 Department of Physics & Astronomy, College of Arts and Sciences, University of Pennsylvania, United States

*sergio.pequito@tudelft.nl

In S1 we provide information about Pearson's correlation. This is followed by an overview on coherence, in S2. Furthermore, S3 gives a brief introduction to the concept of eigen brain analysis which is used as one of the evaluation measures.

S1 Pearson's correlation [1]. Pearson's Correlation is a time-domain similarity measure and provides a relative measure of linear association between two signals. It is given by

$$\rho_{\text{corr}}(x,y)=\frac{\text{cov}(x,y)}{\sqrt{\text{var}(x)\,\text{var}(y)}}=\frac{(x-\bar{x})(y-\bar{y})^T}{\left(\sqrt{(x-\bar{x})(x-\bar{x})^T}\right)\left\{\sqrt{((y-\bar{y})(y-\bar{y})^T)}\right\}} \tag{1}$$

where $\text{cov}(x,y)$ is the covariance between the signals, $\text{var}(x)$ and $\text{var}(y)$is the variance of the signal x and y respectively, and $\bar{x}$ and $\bar{y}$ represent the mean of the respective signals.

The Pearson correlation coefficient ρ_{corr} is scale-invariant and lies in the range from $+1$ to -1. A value closer to 0 implies that there is no linear correlation between two signals. A positive value indicates a positive correlation, that is, both the time series signals tend to be simultaneously greater than their respective means. A negative value implies a negative correlation, that is, the time series tend to fall on opposite sides of their respective means.

S2 Coherence [2]. The spectral coherence also known as *magnitude-squared coherence* assess the correlation between two signals in the frequency-domain. It provides a measure of similarity between the two signals at each frequency and is given by

$$\rho_{\text{coherence}}(x,y)=\frac{|P_{xy}(f)|^2}{P_{xx}(f)P_{yy}(f)} \tag{2}$$

where $P_{xx}(f)$ and $P_{yy}(f)$ are the power spectral densities of two signals respectively, and $P_{xy}(f)$ is the cross power spectral density between the two time series.

The value of the coherence $\rho_{\text{coherence}}$ lies between 0 and 1, 0 indicating no coherence and the value 1 indicates strong coherence between the two time series. Since the coherence is calculated as a correlation between two signals at each frequency, the mean value of the coherence vector thus obtained can be used for further analysis.

S3 Eigen brain analysis. Each ROI from the n ROIs provides us with a time series of resting-state BOLD fMRI. Let $x(k) \in \mathbb{R}^n$ be the vector of $n(=100)$ BOLD signals, where the ith entry $x_i(k)$ correspond to the signal collected at ROI i at the sampling time $k = 1, \ldots, T$. x_k reprsented as $x[k] = [x_1[k] \ldots x_n[k]]^\top$, with $k = 1, \ldots, T$ is the state of the system describing the evolution of the BOLD signal across different regions of brain. Therefore, the systems's state can be modelled as:

$$x(k) = Ax(k-1) + \varepsilon(k), \quad k = 1, \ldots, T \tag{3}$$

where A is the $n \times n$ real matrix describing the coupling between the state variables, whose elements are found by solving a least square optimisation problem [3], and $\varepsilon(k) \in \mathbb{R}^n$ is the approximation error.

The linear time-invariant description of the dynamics of system (Eq 3) can be used to study its dynamical properties using a so-called *eigenmode decomposition*. The eigendecomposition of A provides with the n eigenmodes, that is, the n eigenvalue-eigenvector pairs. These eigenmodes capture the spatiotemporal characteristics of the process. Each eigenmode is represented by an eigenvalue-eigenvector pair (λ_i, v_i) describing the oscillatory dynamics for a specific direction (v_i). The complex eigenvalue (λ_i) captures the frequency of the oscillatory dynamics. Specifically, representing (λ_i) in its polar coordinates $(\theta_i, |\lambda_i|)$ provides the frequency of the oscillation by

$$f_i = \frac{\theta_i}{2\pi}\delta t, \tag{4}$$

where δt corresponds to the sampling frequency. The stability of the process is defined by the absolute of eigenvalue i.e., $|\lambda_i|$. The process is said to be stable if $|\lambda_i| < 1$ and unstable $|\lambda_i| > 1$. The value of $|\lambda_i|$ exactly equals to 1 implies marginal stability.

References

[1] Gibbons JD, Chakraborti S. Nonparametric statistical inference. CRC Press; 2020.

[2] González AG, Rodrıguez J, Sagartzazu X, Schumacher A, Isasa I. Multiple coherence method in time domain for the analysis of the transmission paths of noise and vibrations with non stationary signals. Proceedings of ISMA 2010. 2010;.

[3] Neumaier A, Schneider T. Estimation of parameters and eigenmodes of multivariate autoregressive models. ACM Transactions on Mathematical Software (TOMS). 2001;27(1):27–57.
