# Supplementary figures and images for: Model-based stationarity filtering of long-term memory data applied to resting-state blood-oxygen-level-dependent signal
Source: PLoS One. 2022 Jul 27;17(7):e0268752. doi: 10.1371/journal.pone.0268752 (PMC9328502; doi:10.1371/journal.pone.0268752)

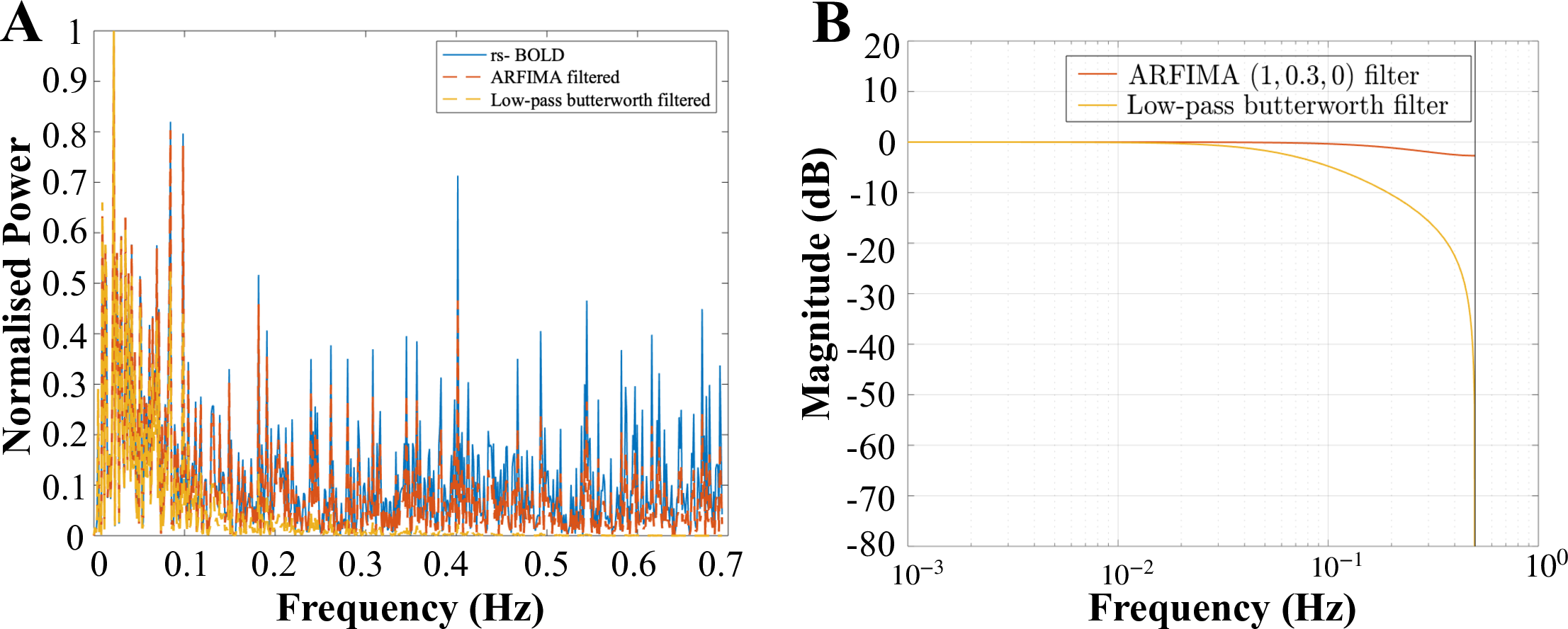

Supplement: S1 Fig — (TIFF) [file pone.0268752.s002.tiff]
