# Supplementary material for: Model-based stationarity filtering of long-term memory data applied to resting-state blood-oxygen-level-dependent signal
Source: PLoS One. 2022 Jul 27;17(7):e0268752. doi: 10.1371/journal.pone.0268752 (PMC9328502; doi:10.1371/journal.pone.0268752)
Supplement: S1 Table — The comparison of these statistics show that the presence of RSNs in each of the identified clusters before and after proposed filtering is similar. (PDF) [file pone.0268752.s003.pdf]

S1 Table. R^2 statistic and p-value of the spatial correlation between the cluster centroid and the identified RSNs. The comparison of these statistics show that the presence of RSNs in each of the identified clusters before and after proposed filtering is similar.

	R^2 Statistic		p-value	
	Before filtering	After filtering	Before filtering	After filtering
Cluster 1	0.1110	0.1114	0.0830	0.0818
Cluster 2	0.4442	0.4407	3.2920 e-10	4.386 e-10
Cluster 3	0.1272	0.1380	0.0443	0.0285
Cluster 4	0.0948	0.0921	0.1496	0.1641
Cluster 5	0.3272	0.3080	1.3301 e-06	4.4098 e-06
