# Supplementary material for: Model-based stationarity filtering of long-term memory data applied to resting-state blood-oxygen-level-dependent signal
Source: PLoS One. 2022 Jul 27;17(7):e0268752. doi: 10.1371/journal.pone.0268752 (PMC9328502; doi:10.1371/journal.pone.0268752)
Supplement: Attachment — Submitted filename: Response To Reviewers.pdf [file pone.0268752.s004.pdf]

Model-based stationarity filtering of long-term memory data applied to resting-state blood-oxygen-level-dependent signal

(Manuscript ID PONE-D-21-22658)

Response Letter

Dear editors and reviewers,

We thank you for investing your precious time and providing us a valuable feedback on our manuscript. We have incorporated all of your feedback in the revised version of our manuscript. In the following parts of this letter, we address the concerns of the reviewers one by one and provide a brief description on how these concerns have been handled in the updated version of the manuscript. We are confident that this letter and the revised version of the manuscript clarify the concerns raised by the reviewers, and we look forward to hearing from you at your earliest convenience.

Sincerely,
Ishita Rai Bansal (on behalf of all the authors)

Associate Editor

(The Associate Editor feedback is highlighted in blue.)

AE.1 We thank you for handling the manuscript and giving us the opportunity to revise and resubmit it.

Comments to the Author

1.) Is the manuscript technically sound, and do the data support the conclusions?
The manuscript must describe a technically sound piece of scientific research with data that supports the conclusions. Experiments must have been conducted rigorously, with appropriate controls, replication, and sample sizes. The conclusions must be drawn appropriately based on the data presented.
Reviewer #1: Yes
Reviewer #2: Yes
Reviewer #3: Partly
Reviewer 3 raised concerns about the preprocessing considered, which has been done and validated by other studies and, therefore, accepted by the community. Furthermore, the reviewer had some concerns about the application of the proposed filter, specifically, if it is performing as it was supposed to. Long story short, we provided evidence that suggests so. In particular, we would like to take the opportunity to highlight that in our manuscript, we added a detailed example where we considered synthetic signals with different frequencies often found in rs-fMRI signals. To these, we added different noises and we showed that it is possible to retrieve the original synthetic signal. Simply speaking, it provides clear evidence that the filter is doing as it was supposed to do.

2.) Has the statistical analysis been performed appropriately and rigorously?
Reviewer #1: Yes
Reviewer #2: Yes
Reviewer #3: I don't know
We would like to emphasise the fact that we have conducted several statistical tests. For example, Kolmogorov–Smirnov test to provide statistical evidence that the proposed solution is able to filter out the effect of high frequency artefacts. Furthermore, it is important to emphasise that without the statistical tests, we would not have been able to support our proposed solution for model-based filtering of preprocessed rs-fMRI signals.

3.) Have the authors made all data underlying the findings in their manuscript fully available?
The PLOS Data policy requires authors to make all data underlying the findings described in their manuscript fully available without restriction, with rare exception (please refer to the Data Availability Statement in the manuscript PDF file). The data should be provided as part of the manuscript or its supporting information, or deposited to a public repository. For example, in addition to summary statistics, the data points behind means, medians and variance measures should be available. If there are restrictions on publicly sharing data—e.g. participant privacy or use of data from a third party—those must be specified.
Reviewer #1: Yes
Reviewer #2: Yes
Reviewer #3: Yes

4.) Is the manuscript presented in an intelligible fashion and written in standard English?
PLOS ONE does not copyedit accepted manuscripts, so the language in submitted articles must be clear, correct, and unambiguous. Any typographical or grammatical errors should be corrected at revision, so please note any specific errors here.

Reviewer #1: Yes
Reviewer #2: Yes
Reviewer #3: Yes

Reviewer 1

(The reviewer feedback is highlighted in blue.)

In "Model-based stationarity filtering of long-term memory data applied to resting-state blood-oxygen-level-dependent signal", Bansal et al. propose a new method to filter unknown source of noise present in resting-state blood-oxygen-level-dependent (BOLD) signal acquires through functional MRI. The proposed method is based on autoregressive fractional integrative moving average (ARFIMA) process. The study is very interesting, well written, and well reported. However, there are certain confusions regarding different parameters' choices, which need clarification by the authors. Specific comments to the authors are given below.

R1.0 First and foremost, we thank the reviewer for his/her time and the invaluable feedback that helped us improve the revised version of the manuscript. We also thank you for the summary and the kind words supporting the quality of our work. In what follows we address in further detail the reviewer's concerns.

1. Materials and Methods
a.) Why are resting-state fMRI (rs-fMRI) time series assumed to be stationary in the study? Based on previous studies resting-state fMRI time series are non-stationary due to presence of various artifacts (Piaggi1, Menicucci et al. 2011, Patel, Kundu et al. 2014, Guan, Jiang et al. 2020, Davey, Grayden et al. 2021). Authors should provide references about the rs-fMRI time series being stationary.

R1.1.a We want to point out that a signal is called stationary if, given a specific model, its statistical properties (i.e. mean, variance and covariance) do not change over time [2]. Usually, the models considered belong to the class of ARMA processes, and these do not yield stationarity. However, models like the proposed ARFIMA may satisfy the stationarity properties. In particular, we would also like to highlight that in order to assess stationarity, the statistical tests are performed on the error between the model and the data being considered. If these follow Gaussian like properties, then we cannot reject the hypothesis that stationarity holds.

b.) What can be the possible advantage of using fractional differencing (in ARFIMA) instead of just differencing (in ARIMA) for filtering? ARIMA may be simpler to implement compared to ARFIMA.

R1.1.b We would like to highlight that the ARFIMA models are a larger class of models that contain the class of ARIMA models, that is obtained when the value of the fractional difference parameter ′d′ is restricted to integers. However, as pointed out in Table 1 in the manuscript, the estimated value of this parameter are not integer values. Hence, we concluded that the ARFIMA models would be more suitable choice for the process we propose.
Additionally, during a diagnoses phase, it is possible to get statistical evidence that the considered ARFIMA models attain the stationarity as discussed in R1.1.a.

c.) The choice of ARFIMA process is used for both stationary non-stationary time series based on the values of the fractional difference 'd' $[-0.5, 0.5]$ for non-stationary case) (Lopes, Olbermann et al. 2004, Box, Jenkins et al. 2016). However, the value of 'd' is estimated from the interval $[0.1, 5.0]$ and the estimated values of 'd' are outside the interval $[-0.5, 0.5]$ for both synthetic and BOLD fMRI time series. The authors should provide the reason of ignoring this difference.

R1.1.c Firstly, we want to thank the reviewer for highlighting an important point that can lead to misunderstanding of the choice of the identified parameters of the model. It is worthwhile to note that the value of fractional difference parameter ′d′ is any real number as pointed out in [6]. However, as mentioned by the reviewer, many studies restrict the value of ′d′ in the interval mentioned which is usually related to the so-called Hurst exponent [3], [8] and may help in finding specific set of factors. Interestingly, 'd' is related with the Hurst exponent by $d = H - 1/2$ [5], which leads to capture any value in the unitary interval, that extends to the

reals by considering the integer integrative component of the model. Thus, we obtain that the 'd' can be any real scalar, which extends the class of ARIMA models to ARFIMA.

d.) It is suggested that authors introduce non-stationarity in the synthetic time series and perform filtering on both stationary and non-stationary synthetic time series using ARFIMA process.

R1.1.d Here, we would like to highlight that we added noise to the synthetic time series to force non-stationarity of the proposed model. At the same time, we appreciate the reviewer's feedback about including the results of filtering the non-stationary synthetic time series using ARFIMA processes. Therefore, in the revised version of the manuscript, we decided to make it more explicit by including a new figure (Figure 3A) which shows the time series of the noisy synthetic BOLD signal.

e.) It is mentioned that differencing is used to remove non-stationarity in the time series (page 4), however, in the study it is applied on rs-fMRI assuming it to be stationary. Authors should comment on this.

R1.1.e It is true that most of the preprocessing removes some of the artefacts but do not gurantee that the models used to consider these preprocessed signals will be stationary. Hence, we propose the usage of ARFIMA models in our research, – see discussion in points R1.1.a and R1.1.b.

2. Results

a.) Why is the frequency range for the synthetic data restricted to 0.1-0.15 Hz? This is the frequency range obtained after the low-pass filtering of the rs-fMRI time series and the study is targeting to look into the frequencies above this range. Keeping this in mind the synthetic data should have higher frequencies included in it, too.

R1.2.a We restricted the synthetic data in the range 0.1-0.15 Hz because we wanted it to mimic the preprocessed rs-fMRI signals (upon which we employ ARFIMA filtering), which can be seen from the power spectrum of rs-fMRI BOLD and synthetic BOLD as shown in Figure 3 and 4, respectively. The higher frequencies in the preprocessed rs-fMRI were removed by the preprocessing steps performed by our collaborators at the University of Pennsylvania, as described in "Section Dataset and Preprocessing" in the manuscript.
Interestingly, it is important to notice that the proposed process in itself is data-driven, and in this particular case, it a low-pass filter as can be seen from the Bode plot characteristic of ARFIMA filter shown in S1 Fig B in the Supplementary Information section of the main manuscript.

b.) What conclusion can be drawn from the result that almost 50% of normalized power spectrum differences (before and after filtering) are not significant?

R1.2.b By considering a model-based filter, we are able to characterize the underlying signal, upon which the noises will be filtered out. The fact that almost 50% remain statistically indistinguishable with a significance level of 0.05 from the other signals, further corroborates the suitability of the proposed model as consequence of the appropriateness of the preprocessing pipeline, to remove some of the unstructured noise.

c.) Why eigenvectors (from eigenmode analysis) are clustered in five clusters?

R1.2.c We would like to thank the reviewer for highlighting our missing justification. Our previous work examined the eigenvector clusters' community organization in-depth across various topological scales. We leveraged a host of different criteria to identify the presence of an optimal topological scale. The non-converging results reveal the multi-scale organization does have a clear optimal topological scale. Therefore, we used the elbow method to identify the subjective optimal resolution in our previous and current work to illustrate our main point. We removed these analyses from this manuscript for brevity. Nevertheless, we agree with the reviewer that it is important to report these details. Therefore we provide a new reference in the results section, to direct the interested reader to our previous manuscript, lines 265-266.

3. Discussion

a.) The results report attenuation in high frequencies after filtering with ARFIMA process and amplification in few cases. What are the relative percentages of attenuations and amplifications? Can something be concluded about the reason of attenuation or amplification? What does it mean by' the proposed filter seems to attenuate/amplify the higher frequencies, when required? It is not clear 'required' by what?

R1.3.a We have determined the parameters of the proposed model-based filters through data, and the behavior observed is case dependent, as the following scenarios exemplify. In particular, from the magnitude Bode plots of the transfer function associated with the designed filters for three different ROIs as shown in Figure 1 below.

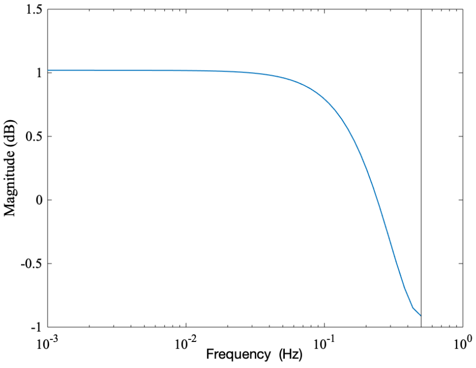

(a)

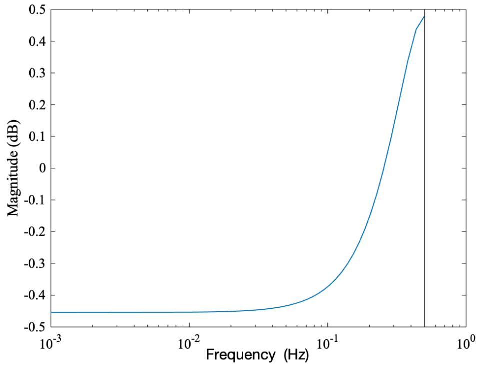

(b)

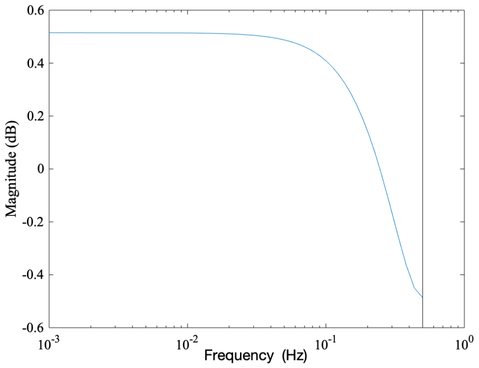

(c)

Fig. 1: Magnitude bode plot of the transfer function associated with designed filters for three different ROIs. The three plots shown in (a), (b) and (c) for ROIs 2, 10 and 49 respectively illustrates diverse behaviour of the filter of amplification/attenuation in higher frequencies.

4. Minor points

a.) The form of the fractional differencing mentioned in (4) is specifically for the case d >-1 (Xiu and Jin 2007), which is not mentioned specifically in the study.

R1.4.a We have explicitly indicated this in the revised version of the manuscript, as well as its generalization. Specifically, it now reads as follows:
"The difference operator can be represented in terms of the gamma function by solving its binomial expansion when $d > -1$ [7] as given in Eq. 4 in the manuscript. Notwithstanding, we can generalize for any value of d by noticing that the operator $(1-B)$ is linear. Specifically, for any $d' \in \mathbb{Z}$ and $d > -1$, we can have $(1-B)^{d'+d} = (1-B)^{d'}(1-B)^{d}$, where the first term is a difference equation and the second is given in Eq.4."

b.) Caption of the Figure 5 is inverted.

R1.4.b We looked into the caption of figure 5 (i.e. figure 6 in the revised version of the manuscript). It looks correct to us.

c.) Figure 9 is inverted.

R1.4.c We appreciate reviewer's concern and double checked the figure 9 (now figure 10 in revised manuscript). However, as in the previous point, it looks correct to us.

Reviewer 2

(The reviewer feedback is highlighted in blue.)

This manuscript describes the unique application of the autoregressive fractional integral moving average (ARFIMA) filter to resting-state BOLD synthetic and real data. The manuscript reports that ARFIMA can attenuate higher frequencies without impacting on traditional FC measures. Furthermore, they demonstrate filtered data has improved "stability" as estimated using eigenmode analysis of directed connectivity.
This method has merit for filtering out true "noise" from the fMRI and would be more nuanced alternative to traditional spectral filters which are often used in rsfMRI pre-processing. However, I think the clarity of the manuscript undermines the import of the topic.

R2.0 We want to thank the reviewer for taking out his/her time and providing us the invaluable feedback that helped us improve the revised version of the manuscript. We try to address reviewer's concerns in the following sections.

*1.) It is not explicitly stated, but I *think* that the rationale for the ARFIMA filter in this context is that it will retain signal with a long-term autocorrelation structure presumed to be neural signal, and filter out noise without this autocorrelation structure. This seems like a reasonable motivation to me; however, I feel that the manuscript lacks a clear and explicit exposition of this motivation. It is attempted in the second-to-last paragraph of the introduction but it could be a lot clearer and more explicit. On first read, it feels like the motivation for ARFIMA was largely that it has never been using in this context before...*

R2.1 We appreciate the reviewer's feedback for providing such a clear understanding of our manuscript. In order to address the reviewer's concern, we have revised the introduction of the manuscript to be more explicit and clear. Specifically, in the context of this concern, refer lines 35 - 50 in the revised version of the main manuscript.

2.) The term "denoising" is often loosely used in the rsfMRI literature to refer to the removal of both unwanted structured artefacts and/or unstructured "noise" in the more traditional sense. The authors seek to clarify that their proposed method deals only the latter (filtering unstructured noise), and not structured artefact, nonetheless this needs to be emphasised as it has the potential to be missed by the reader which will result in confusion for readers that are used to the more liberal interpretation.

R2.2 In order to avoid the possible confusion about whether denoising in our context mean removal of structured artefacts or unstructured noise, we have revised the introduction section of the manuscript to emphasize the focus on the unstructured noise – refer lines 35 - 50 in the revised version of the main manuscript.

3.) You have chosen to include GSR within the definition of "minimally" pre-processed. This is a controversial choice that not all in the community would agree with. I don't think the inclusion or exclusion of the global signal, which is a structured signal, would impact the performance of ARFIMA, but this should be minimally addressed, and ideally even demonstrated...

R2.3 We would like to thank the reviewer for highlighting the controversy over GSR. We fully agree that it is essential to disclose to the readers all the preprocessing steps with likely outsized effects on the modeled dynamics and the results. Currently, almost all methods such as GSR and PCA-based methods that aim to remove the global significant physiological noise (e.g., cardiac and respiratory noise) suffer from the same limitations. Therefore, having weighed the potential drawbacks of GSR against the major concerns regarding the significant global artifacts such as the cardiac and respiratory noise, we adopted the GSR preprocessing step. Nevertheless, we acknowledge that it is beneficial for future work to investigate the spectral profile of the global signal and the impact of GSR on the estimated system and inputs' spectral characteristics. Following the reviewer's suggestion, included lines 405-417

in the methodological consideration and limitation section in main manuscript about the GSR controversy. Additionally, we also decided to remove the definition of minimally pre-processed from our manuscript to avoid further misunderstandings.

4.) ICA-FIX seeks to remove both structured and unstructured noise from BOLD therefore ARFIMA is being presented with BOLD data that already has attenuated unstructured noise. It would be interesting to use ICA to remove only the structured noise, and then see how well ARFIMA performs on the unstructured noise (without the prior attenuation). It seems plausible to me that ARFIMA may do better on unstructured noise than ICA-FIX and could/should potentially be used in conjunction with FIX.

R2.4 We thank the reviewer for raising an interesting point regarding ICA-Fix and removal of the noise component. As we did not have any prior hypotheses on the neural origin of the unstructured noise we used ICA-FIX to remove both structured and unstructured noise. In addition, using the same preprocessing pipeline as our prior studies [1] allows for continuity and more direct comparison across different methods explored by our team. Nevertheless, we fully agree with the reviewer that understanding how this preprocessing step can impact our results is highly relevant. Therefore, we decided to highlight this important point in the main manuscript, lines 418-423.

5.) Figure 3 presents metrics for the synthetic BOLD. It would be valuable to the see the same metrics for the pre-processed bold alongside the synthetic. Furthermore, I would prefer to also see an example time-course from both the BOLD and synthetic data.

R2.5 We thank the reviewer for providing this feedback. We also believe that it is valuable to add this figure in the main manuscript which weighs the pros and cons of the proposed methodology. Hence, we added the suggested figure in the manuscript, lines 189-190.

6.) The non-symmetric colorbars in figures 7 and 8 make it harder to interpret the matrices.

R2.6 We tried different representations and concluded that the one we used seemed to be the easiest to assess the variations in the functional connectivity matrices, statistics to reflect both the mean and the variance.

7.) Table 2 would be easier to interpret as a bar plot.

R2.7 We would like to thank reviewer for his/her suggestion. We tried plotting Table 2 as a bar plot, however, it made it more difficult to interpret. Specifically, by representing the values of 'd' through table, we wanted to highlight the distinct values of fractional difference parameter 'd' which the colorbar does not reflect properly.

8.) I found it odd that new figures were introduced in the discussion.

R2.8 We thank the reviewer once again for his/her feedback. In the revised version of the manuscript, we have added these figures in the supplementary information. We would like to point that the role of these figures and claims in the discussion is to provide evidence that some additional tests and observations are backed up by experimental results and a sound methodology.

Reviewer 3

(The reviewer feedback is highlighted in blue.)

The authors conduct a potentially interesting fMRI filtering study that aims to address the problem of unstructured noise. Unfortunately, the manuscript is let down by issues of presentation and I found the methods confusing. I list several analyses that would need to be performed for evaluation before I would recommend publication.

R3.0 We are grateful to the reviewer for his/her feedback that helped us greatly improve the manuscript and appreciate him/her for taking out time to evaluate our manuscript. In the following parts, we address reviewer's concern point by point.

1. Major Concerns:
a.) Manuscript organization for Peer Review: Today most manuscripts are reviewed on a computer as an electronic PDF file. As a result, it is very problematic to put the figures at the end of the document, as this requires the overworked peer reviewer to scroll back and forth in the document. Figures and legends should be embedded within the manuscript at logical locations and high-quality PDF conversion for figures should be handled by the authors (i.e., don't rely on poorly configured journal/publisher websites) to ensure that the manuscript is presentable to the peer reviewers. This allows the reviewer to focus on your science and not problems with the organization. I have scrolled through the document once, but may have missed things.

R3.1.a We understand the reviewer's concern about the organisation of manuscript. However, we would like to point out that it is the requirement of the journal that the figures and manuscript is submitted in two separate files.

b.) It appears that the authors are starting from the data that has not been cleaned by spatial ICA+FIX. This is incorrect. If they are interested in working on the unstructured noise problem, they should work with data containing only neural signal and unstructured noise. Later it looks like spatial ICA+FIX was in fact used. I would not use the terminology, "minimal preprocessing," as most folks take that to mean the outputs of the HCP's spatial preprocessing pipelines (i.e., Glasser et al., 2013 Neuroimage), before temporal preprocessing (like linear trend removal or sICA+FIX).

R3.1.b We appreciate the reviewer's feedback that the term minimal preprocessing could lead to confusion among the readers. Therefore, following the reviewer's suggestion, we have replaced the term minimal preprocessing with preprocessing in the main manuscript.

c.) What is the justification for using global signal regression in this study? Glasser et al., 2018; 2019 Neuroimage shows that the global signal is a roughly 50/50 mix of neural signal and global respiratory artifact.

R3.1.c We thank the reviewer for addressing this concern and we fully agree that there are trade offs to GSR. Currently almost all methods like GSR or PCA-based that aim to remove global significant physiological noise (like respiratory and cardiac) suffer from the same limitation as mentioned by Glasser *et al.*. We totally understand the drawbacks of GSR, however, it cannot be beat for removing motion signal from data, which is likely critical for your analyses. At the same time, we also acknowledge that it would be beneficial to understand the impact of GSR on estimated system and its inputs. Therefore, agreeing with reviewer's suggestion, we decided to highlight this important point in the main manuscript, see lines 405-417.

d.) It is said that the "98 subjects with the least movement artifacts" were used. Why? sICA+FIX should have already addressed the spatially specific effects of head motion.

R3.1.d We appreciate reviewer's concern but would like to point out that no method is perfect for totally removing the noise. Hence, we considered "98 subjects with the least movement

artifacts" to increase our odds of dealing with the most important rs-fMRI artifact by starting from subjects with low motions. That said, the sample size considered is more than sufficient to establish the statistical validity of the obtained findings.

e.) I must admit I did not follow the filtering procedure at all. Probably what the authors need to show to convince me that it is not doing something bad is all of the following: a) Show the effect of the filtering on i) The signal ICA component timeseries and ii) The unstructured noise timeseries after performing the filtering on the overall timeseries. This could be accomplished by taking the filtered timeseries and spatially regressing the signal sICA component spatial maps into the filtered timeseries to generate filtered component timeseries. These could then be compared with the originals to ensure that the filtration approach is not affecting the neural signal. Also, the overall unstructured noise variance after filtration should be compared to that before filtration. The unstructured noise variance can be obtained by regressing out the signal ICA components and taking the variance of the residuals. b) Show the difference in task fMRI activation betas before and after this filtration procedure. The betas should not change much and ideally the pattern of change should match the overall activation pattern, but the zstats might improve (though they should be corrected using mixture modelling, such as that available in melodic, before and after filtration).

R3.1.e We would like to take the opportunity to emphasize the fact that in order to guarantee that the proposed filter is consistent with its results, we generated a synthetic signal with properties similar to the rs-fMRI signal and reported the results of the application of proposed model on the noisy synthetic signal to retrieve the clean signal. The obtained results highlight that the proposed method is able to perform as desired.
Additionally, it is also important to notice that the we considered rs-fMRI signals for our story because we need enough data to estimate the signal for which the model is stationary. However, in task based fMRI signals, the response driven by external factors is short lived which is not captured throughout the model. Hence, we implemented the proposed filter on the resting-state signals.

2. Minor points

a.) Line 18: Marcus et al had errors in the formulas. Use Glasser et al., 2018 Neuroimage Supplemental topic #3 for more accurate measures.

R3.2.a We had the opportunity to revise the entire document for inconsistencies and typos. Unfortunately, we could not find the reference to the equations mentioned by the reviewer in [4].

b.) The spatial preprocessing of Glasser et al., 2013 Neuroimage should be mentioned.

R3.2.b In line 417 in the manuscript, we have added the reference as suggested by the reviewer to highlight the importance of different types of preprocessing pipelines on rs-fMRI data.

c.) It is not said whether volume or CIFTI grayordinates data is used here. CIFTI is recommended.

R3.2.c We used the CIFTI surface preprocessing, including the parcellation of the time series in fsLR32k space.

References

[1] Arian Ashourvan, Sérgio Pequito, Maxwell Bertolero, Jason Z Kim, Danielle S Bassett, and Brian Litt. External drivers of BOLD signal's non-stationarity. *bioRxiv*, 2021.

[2] George EP Box, Gwilym M Jenkins, Gregory C Reinsel, and Greta M Ljung. *Time Series Analysis: Forecasting and Control*. John Wiley & Sons, 2015.

[3] YangQuan Chen, Rongtao Sun, and Anhong Zhou. An improved Hurst parameter estimator based on fractional Fourier transform. *Telecommunication Systems*, 43(3):197–206, 2010.

[4] Matthew F Glasser, Timothy S Coalson, Janine D Bijsterbosch, Samuel J Harrison, Michael P Harms, Alan Anticevic, David C Van Essen, and Stephen M Smith. Using temporal ICA to selectively remove global noise while preserving global signal in functional MRI data. *NeuroImage*, 181:692–717, 2018.

[5] Timothy Graves, Robert Gramacy, Nicholas Watkins, and Christian Franzke. A brief history of long memory: Hurst, Mandelbrot and the road to ARFIMA, 1951–1980. *Entropy*, 19(9):437, 2017.

[6] Abdul Aziz Karia, Imbarine Bujang, and Ismail Ahmad. Fractionally integrated ARMA for crude palm oil prices prediction: case of potentially overdifference. *Journal of Applied Statistics*, 40(12):2735–2748, 2013.

[7] Jin Xiu and Yao Jin. Empirical study of ARFIMA model based on fractional differencing. *Physica A: Statistical Mechanics and its Applications*, 377(1):138–154, 2007.

[8] Xianming Ye, Xiaohua Xia, Jiangfeng Zhang, and Yangquan Chen. Effects of trends and seasonalities on robustness of the Hurst parameter estimators. *IET Signal Processing*, 6(9):849–856, 2012.
